# Disruption of TGF-β Signaling Improves Ocular Surface Epithelial Disease in Experimental Autoimmune Keratoconjunctivitis Sicca

**DOI:** 10.1371/journal.pone.0029017

**Published:** 2011-12-14

**Authors:** Cintia S. De Paiva, Eugene A. Volpe, Niral B. Gandhi, Xiaobo Zhang, Xiaofen Zheng, John D. Pitcher, William J. Farley, Michael E. Stern, Jerry Y. Niederkorn, De-Quan Li, Richard A. Flavell, Stephen C. Pflugfelder

**Affiliations:** 1 Ocular Surface Center, Department of Ophthalmology, Cullen Eye Institute, Baylor College of Medicine, Houston, Texas, United States of America; 2 Shanxi Eye Hospital, Taiyuan, China; 3 Department of Ophthalmology, University of Texas Southwestern Medical Center, Dallas, Texas, United States of America; 4 Howard Hughes Medical Institute and Department of Immunobiology, Yale University School of Medicine, New Haven, Connecticut, United States of America; University Medical Center Freiburg, Germany

## Abstract

**Background:**

TGF-β is a pleiotropic cytokine that can have pro- or anti-inflammatory effects depending on the context. Elevated levels of bioactive TGF-β1 in tears and elevated TGF-β1mRNA transcripts in conjunctiva and minor salivary glands of human Sjögren's Syndrome patients has also been reported. The purpose of this study was to evaluate the response to desiccating stress (DS), an experimental model of dry eye, in dominant-negative TGF-β type II receptor (CD4-DNTGFβRII) mice. These mice have a truncated TGF-β receptor in CD4^+^ T cells, rendering them unresponsive to TGF-β.

**Methodology/Principal Findings:**

DS was induced by subcutaneous injection of scopolamine and exposure to a drafty low humidity environment in CD4-DNTGFβRII and wild-type (WT) mice, aged 14 weeks, for 5 days. Nonstressed (NS) mice served as controls. Parameters of ocular surface disease included corneal smoothness, corneal barrier function and conjunctival goblet cell density. NS CD4-DNTGFβRII at 14 weeks of age mice exhibited a spontaneous dry eye phenotype; however, DS improved their corneal barrier function and corneal surface irregularity, increased their number of PAS+ GC, and lowered CD4^+^ T cell infiltration in conjunctiva. In contrast to WT, CD4-DNTGFβRII mice did not generate a Th-17 and Th-1 response, and they failed to upregulate MMP-9, IL-23, IL-17A, RORγT, IFN-γ and T-bet mRNA transcripts in conjunctiva. RAG1KO recipients of adoptively transferred CD4+T cells isolated from DS5 CD4-DNTGFβRII showed milder dry eye phenotype and less conjunctival inflammation than recipients of WT control.

**Conclusions/Significance:**

Our results showed that disruption of TGF-β signaling in CD4^+^ T cells causes paradoxical improvement of dry eye disease in mice subjected to desiccating stress.

## Introduction

TGF-β is a pleiotropic cytokine that can have pro- or anti-inflammatory effects depending on the context. It regulates various biologic processes such as embryonic development, cell proliferation and differentiation, extracellular matrix synthesis, immune response, inflammation, and apoptosis [1]. TGF-β1 is produced by the human lacrimal gland (LG) and corneal and conjunctiva epithelia and has been detected in tears [2], [3]. Elevated levels of bioactive TGF-β1 in tears and elevated TGF-β1mRNA transcripts in conjunctiva and minor salivary glands of human Sjögren's Syndrome (SS) patients has also been reported [4]–[7]. In the spontaneous model of SS in the CD25KO mice, destruction of LG architecture is accompanied by increased levels of TGF-β1 mRNA [8]. On the other hand, if TGF-β is absent or if TGF-β signaling is impaired at birth, mice develop intense T cell lymphoproliferation and autoimmunity with aging, leading to death. As a prime example, TGF-β1KO mice die a few days after birth due to massive and generalized lymphocytic infiltration [9], and their lacrimal and salivary glands are heavily infiltrated with CD4^+^T cells [10] mimicking SS. Another example is a strain containing a conditional TGF-β receptor knockout in mammary glands that are born normal, but die at 4–5 weeks of age due to severe multifocal inflammation in salivary and mammary glands and heart [11].

Because of its diverse functions, regulation of TGF-β activity maybe a check-point in the immune system. It has long been recognized that TGF-β has an inhibitory effect on immune response by a variety of mechanisms.[12] Not only does it block T and B-cells proliferation, but it has also been found to promote regulatory-T cell (Treg) differentiation and activity [12]–[14]. At the same time, TGF-β is critical to the induction of Th-17 cells [15]–[17].

The transgenic mice expressing a dominant negative truncated form of the TGF-β receptor type II under the CD4 promoter (rendering their CD4^+^ T cells unresponsive to TGF-β) have been used to evaluate the effects of disrupted TGF-β signaling in T cells. They develop autoimmunity characterized by inflammatory cell infiltration of multiple organs, including colon and lung, and may suffer early beginning at 12–14 weeks of age [18], [19].

Although SS is indeed an autoimmune disease of exocrine glands, the work of our group and others indicates there is also a primary autoimmune reaction in the conjunctiva and cornea that when supressed by local antiinflammatory therapy will significantly improve corneal epithelial barrier function and conjunctival goblet cell density [20]–[25]. Therefore, the conjunctiva and corneal disease are as relevant as the LG for clinical manifestations of the disease. Evidence suggests that effector CD4^+^ T cells contribute to the tear dysfuntction that develops in this condition. Inflammation in the lacrimal glands, cornea, and conjunctiva, which results in decreased tear production, and conjunctival goblet cell loss, has been induced by transferring CD4^+^ T cells from mice subjected to experimental dry eye to T-cell–deficient nude mice that have not been exposed to desiccating stress [26]. These findings indicate that CD4^+^ T cells are directly involved in the pathogenesis of dry eye. We have previously reported that our desiccating stress model elicits an increase of TGF-β1 mRNA and protein in conjunctiva and a concomitant increase in IL-17A [6]. In that study, we found that IL-17A disrupts corneal barrier function and increases expression of matrix metalloproteinases. In an animal model of EAE, where IL-17A cells were found to be pathogenic, CD4-DNTGFβRII mice failed to generate Th-17 cells and failed to develop disease [12].

The purpose of this project was two fold: first to investigate time-related changes in DNTGFBRII mice and second, to investigate their response to desiccating stress, an animal model of dry eye.

## Results

### Spontaneous Autoimmune Inflammation of the Ocular Surface mucosa in Aged CD4-DNTGFβRII mice

CD4-DNTGFβRII mice are phenotypically normal until 6∼8 weeks of age, when they start developing spontaneous wasting syndrome and autoimmunity and die around 12∼16 weeks of age, mainly due to colitis [18]. Because absence of TGF-β from birth always correlates with development of autoimmunity, we evaluated the effects of aging on surface inflammation in CD4-DNTGFβRII aged 4, 8 and 14 weeks and compared them to WT mice of similar ages.

We observed that CD4-DNTGFβRII mice had increased CD4+T cell infiltration in the conjunctiva epithelia that was accompanied by increased expression of Th-related cytokine mRNA transcripts in the cornea (IFN-γ; 2.51±0.36 vs. 1.00±0.27 relative fold, P<0.05) and conjunctiva (IFN-γ and IL-17A; 2.73 ±0.29, 3.75±0.50 vs.1.00±0.39, 1.00±0.68 relative fold, respectively, P<0.05 for both) compared to WT littermates of the same age.

Enlargement of lymphoid organs (cervical lymph nodes and spleens) in CD4-DNTGFβRII mice was observed to be greater at 8 weeks than 14 weeks of age; however, we did not observe significant colitis or loose stools and 14-week old mice did not appear clinically dehydrated. All mice subjected to desiccating stress at 14 weeks of age completed the study. Some CD4-DNTGFβRII mice at 14 weeks of age were smaller than their WT littermates.

**Figure 1:**
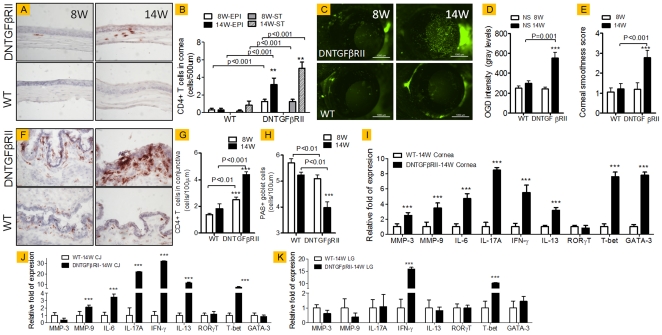
Age-related changes in wild-type (WT) and DNTGFBRII mice, from 8 to 14 weeks of age (8W and 14W, respectively). **A.** Representative images of cornea sections immunostained for CD4 (in red) used to generate CD4 counts in B, in corneal epithelium (EPI) and corneal stroma (ST) in wild-type and DNTGFβRII mice at 8 and 14 weeks of age (8W,14W). Bar charts show mean ± SD of two independent experiment (final n = 5 for each group). **C.** Representative images of OGD corneal staining used to generate OGD intensity score in **D.** Bar charts show mean ± SD of three independent experiments (final n = 12 for each group). **E.** Corneal smoothness score. Bar charts show mean ± SD of three independent experiments (final n = 13 for each group). **F.** Representative images of conjunctiva sections immunostained for CD4 (in red) used to generate CD4 counts in **G.** Bar charts show mean ± SD of three independent experiments (final n = 5 for each group). **H.** PAS+ conjunctival goblet cells counts. Bar charts show mean ± SD of three independent experiments (final n = 5 for each group). **I–K.** Relative fold of expression in cornea (I), conjunctiva, (CJ, in J) and lacrimal gland (LG, in K) using the WT-8W as the calibrator. Bar charts show mean ± SD of three independent experiment (final n = 8 for each group). In graphs B, D,E, G, H **P<0.01;*** P<0.001 indicate within strain comparison In I–K graphs,*** P<0.001 indicates interstrain comparison.

In cornea sections stained for CD4, (Figure 1A) we observed significantly increased numbers of CD4^+^T cells present in both epithelium and cornea stroma (Figure 1B). Young CD4-DNTGFβRII mice also showed increased permeability to the fluorescent molecule OGD (Figure 1C), used as a measure of corneal barrier function compared to WT at the same age, with a spontaneous, significant worsening of OGD staining scores at 14 weeks (Figure 1D). Corneal regularity was normal in both WT and CD4-DNTGFβRII mice at 8 weeks, but an increase in irregularity was noted in CD4-DNTGFβRII at 14 weeks of age (Figure 1E).

In conjunctival sections, we also noted increased CD4+T cell infiltration in conjunctiva epithelium in young CD4-DNTGFβRII, preferentially affecting the goblet cell rich area of the conjunctiva (Figure 1F–1G). Conjunctival PAS+ cells in CD4-DNTGFβRII mice were decreased at 8 weeks, and further decrease was observed with aging at 14 weeks of age (Figure 1H).

Taken together, these findings indicate an accumulation of pathogenic CD4^+^ T cells in the cornea and conjunctiva, inducing a spontaneous dry eye like phenotype characterized by increased corneal irregularity, disrupted corneal barrier function and reduced numbers of filled conjunctival goblet cells in the conjunctiva.

Spontaneous Th-1 and Th-2 differentiation in vitro of CD4^+^ isolated from CD4-DNTGFβRII mice and escape from natural T regulatory cells have been reported previously [19]. We hypothesized that spontaneous differentiation of Th lines including, Th-1, Th-2 and Th-17 may occur on the ocular surface of this strain. To investigate the phenotype of the ocular surface infiltrating cells at 14 weeks of age, we collected and processed cornea, conjunctiva and lacrimal gland for gene analysis.

We observed that 14w-old CD4-DNTGFβRII mice had increased levels of MMP-3, MMP-9, IL-6, IL-17A, IFN-γ and IL-13 in corneal epithelium compared to WT mice of similar age. Transcription factors T-bet and GATA-3 were also found to be elevated in corneal epithelium. Increased expression of MMP-3 and MMP-9 has been found to correlate with corneal epithelial disease [6], [22], [23], [27], [28].

In conjunctiva, we found a significant increase in MMP-9, IL-6, IL-17A, IFN-γ, IL-13 and T-Bet mRNA transcripts. However, LG tissues showed increased expression of only IFN-γ and T-bet.

Taken together, these findings indicate that there is an accumulation of cytokine-producing CD4^+^T cells on the ocular surface with aging.

### Disruption of TGF-β Signaling Improves Dry Eye Disease

**Figure 2:**
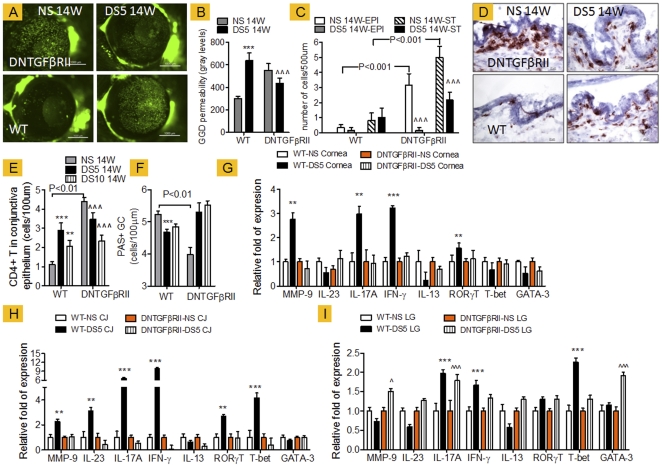
Desiccating stress-induced changes in wild-type (WT) and DNTGFβRII mice at 14 weeks of age (14W). Desiccating stress was induced for 5 days in both strains (DS5); nonstressed mice served as controls (NS). **A.** Representative images of OGD corneal staining used to generate OGD intensity score in **B.** Bar charts show mean ± SD of three independent experiments (final n = 12 for each group). **C.** CD4 counts in corneal epithelium (EPI) and corneal stroma (ST). Bar charts show mean ± SD of three independent experiments (final n = 5 for each group). **D.** Representative images of conjunctiva sections immunostained for CD4 (in red) used to generate CD4 counts in **E**, in conjunctiva epithelium Bar charts show mean ± SD of three independent experiments (final n = 5 for each group). **F.** PAS+ conjunctival goblet cells counts. Bar charts show mean ± SD of three independent experiments (final n = 5 for each group). **G–I.** Relative fold of expression in cornea (I), conjunctiva, (CJ, in J) and lacrimal gland (LG, in K) using the WT-NS of each strain as the strain-calibrator. Bar charts show mean ± SD of three independent experiments (final n = 8 for each group). **P<0.01;*** P<0.001 indicate within WT comparison (NS vs. DS5) ∧ P<0.05;∧∧∧P<0.01 indicates DNTGFBRII comparison (NS vs. DS5).

We hypothesized that the initiation of the immune response that generates pathogenic CD4^+^T cells and contributes to the dry eye phenotype is dependent on desiccating-stress induced TGF-β1 production and not on constitutive levels of TGF-β1. To test this hypothesis, we challenged CD4-DNTGFβRII at 14 weeks of age and their WT littermates by subjecting them to experimental desiccating stress. Our findings are summarized in Figure 2.

After desiccating stress for 5 days, WT mice showed an increase in OGD staining, cornea irregularity (data not shown), increased CD4^+^ T cell infiltration in the conjunctiva and a decrease in PAS+ goblet cells compared to nonstressed WT mice. Paradoxically, DS improved corneal barrier function and corneal surface irregularity, increased the number of conjunctival PAS+ cells, and lowered CD4^+^ T cell infiltration of the conjunctiva in CD4-DNTGFβRII mice. The number of CD4^+^ T cells in the corneal epithelium and stroma also decreased after DS in the CD4-DNTGFβRII mice (Figure 2A–F).

In contrast to WT mice, CD4-DNTGFβRII mice were unable to generate a Th-17 and Th-1 response, and they failed to upregulate Th-17 and Th-1 related genes in cornea (Figure 2G). In conjunctiva, CD4-DNTGFβRII mice failed to upregulate MMP-9, IL-23, IL-17A, RORγT, IFN-γ and T-Bet mRNA transcripts (Figure 2H). In lacrimal gland, we found upregulation of both IL-17A and IFN-γ transcripts, indicating generation of Th-1 and Th-17 response after DS in the LG maybe a TGF-β independent.

Taken together, these results indicate that the murine dry eye phenotype observed in our desiccating stress model is highly dependent on DS-induced TGF-β1 production.

### TGF-β modulates proliferation of T cells after encountering ocular surface epithelium

**Figure 3:**
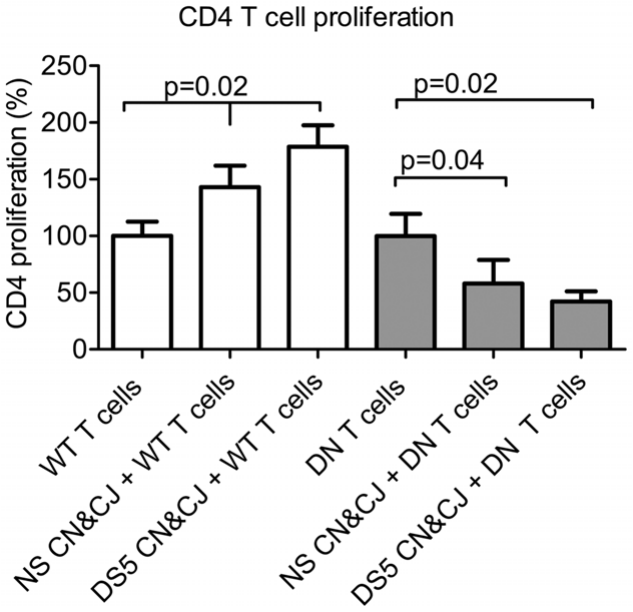
CD4+ T cell proliferation. Proliferation of CD4^+^ T cell in CD4^+^ T cells co-cultured in the presence of cornea and conjunctival tissues (CN and CJ, respectively) before (nonstressed [NS]) or after desiccating stress for 5 days [DS5] from wild-type (WT) and CD4-DNTGFβRII mice (DN). Bar charts show mean ± SD of two independent experiments (final n = 5 for each group).

To evaluate the paradoxal effect of DS in CD4-DNTGFβRII mice, which showed lower T conjunctival cell infiltration after DS, we used a co-culture system where CD4^+^ T cells isolated from spleens and cervical lymph nodes of WT and CD4-DNTGFβRII mice were used as responders and cornea and conjunctiva explants obtained from NS and DS5 mice as stimulators [29]. CD4 proliferation was evaluated using the MST assay. CD4^+^ T cells in this co-culture system have been shown to proliferate after encountering corneal and conjunctiva from DS mice [29]. As seen in Figure 3A, CD4-DNTGFβRII CD4^+^ T cells failed to proliferate in the presence of DS5 explants for 4 days, in contrast to WT CD4^+^ T cells, suggesting that the CD4^+^ cell population from CD4-DNTGFβRII mice may have fewer pathogenic effector cells that are dependent on TGF-β for sustained proliferation.

### TGF-β prevents migration of CD4+ T cells to the conjunctiva

Another possibility for lower T cell infiltration in the conjunctiva CD4-DNTGFβRII mice is that these infiltrating CD4^+^ T cells have impaired migration. Migration of T cells into the cornea and conjunctiva requires a coordinated effort of chemokines, and chemokine receptors to drive the specific cells to the tissue and MMPs to facilitate their migration into tissues. CCR6 is a newly indentified chemokine receptor that is highly expressed on the surface of Th-17 cells and therefore has been used as a marker of Th-17 inflammation [30]. CCR5 and CXCR3, on the other hand, have been found to identify Th-1 cells [30]. TGF-β has been reported to regulate the expression of these chemokine receptors [31].

**Figure 4:**
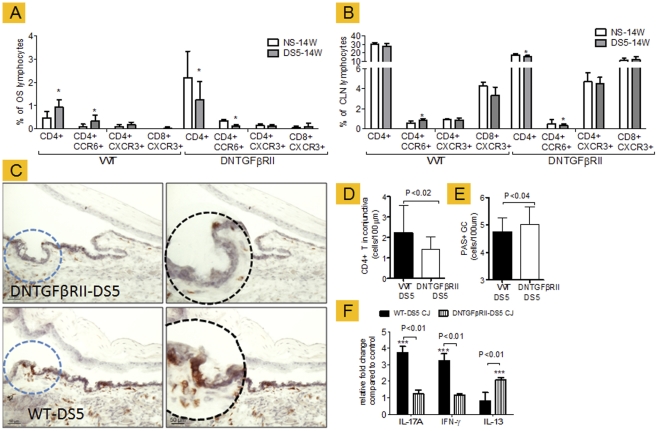
Expression of chemokine receptors and adoptive transfer results in RAG1KO mice. **A–B** Flow cytometry analysis of freshly isolated cells from ocular surface (OS, in A) and cervical lymph nodes (CLN, in B) from wild-type (WT) and DNTGFβRII mice before (nonstressed, NS) and after 5 days of desiccating stress (DS5).*P<0.05, within strain comparison. Bar charts show mean ± SD of three independent experiments (final n = 4 for each group for OS, final n = 6 for each group for CLN). **C.**Conjunctival sections stained for CD4^+^ T cells (in red) of RAG1KO recipient mice that received CD4+ cells isolated from WT and DNTGFβRII DS5 mice. Note CD4+T cell infiltration in goblet cell rich area of conjunctiva in WT-DS5 recipient mice. The black dotted circle is a higher magnification of area demarcated by blue dotted circle. Original magnification: 20×. Scale bar = 50 µm. **D and E**. Conjunctival CD4+T cell **(in D)** and PAS+ conjunctival goblet cells **(in E)** counts in RAG1KO recipient mice that received cells from CD4+ cells isolated from WT and DNTGFβRII mice after 5 days of desiccating stress (DS5). Bar charts show mean ± SD of three independent experiments (final n = 5 for each group) **F**.Relative change of IL-17A, IFN-γ and IL-13 mRNA transcripts in conjunctiva of RAG recipients. Asterisks indicate comparison to strain-specific controls. Bar charts show mean ± SD of two independent experiments (final n = 6 for each group).

To evaluate the expression of chemokine receptors, we performed flow cytometry analysis in freshly isolated cells from ocular surface and cervical lymph nodes from WT and CD4-DNTGFβRII mice, before and after desiccating stress. CD4 and CD8 cells were dual labeled for CCR6 and CXCR3. Our results are presented in Figure 4. We observed that desiccating stress induced an influx of CD4^+^ T cells in the conjunctiva of WT mice and a significant increase of CD4+CCR6+ cells, while both cell types decreased in the DNTGFBRII mice (Figure 4A). Similar results were found in the CLN of both strains (Figure 4B).

Our previous published studies showed that inflammation and epithelial disease in the lacrimal glands, cornea, and conjunctiva can been induced by transferring CD4^+^ T cells from mice subjected to experimental DS to T-cell-deficient mice that have not been exposed to DS [26]. Because this adoptive transfer model offers the possibility to evaluate the migration of systemic transferred “eye” primed pathogenic T cells and the induction of disease in an immunodeficient host, we performed adoptive transfer using CD4^+^ cells isolated from DS5 WT and CD4-DNTGFβRII and evaluated goblet cell density and CD4^+^ T cell infiltration in the conjunctival epithelium. We observed that RAG1KO mice that received CD4^+^ adoptively transferred from CD4-DNTGFβRII DS5 mice had lower CD4^+^T cell infiltration and higher numbers of PAS+ GC in the conjunctiva than WT mice (Figure 4C–E). CD4^+^ T cells from DS5 donor mice localized in the goblet cell rich area of the conjunctiva (Figure 4C, insets). We also observed that CD4-DNTGFβRII DS5 RAG recipient mice had lower IL-17A, IFN-γ and higher IL-13 mRNA transcripts than RAG1KO recipient mice which received cells from WT DS5 control mice (Figure 4F).

These results indicate that TGF-β is critical to generation, migration and survival of Th-17 eye-primed cells.

## Discussion

Our findings indicate TGF-β has at least two different roles on the ocular surface: 1) maintenance of homeostasis and 2) generation of an immune response to stresses, such as desiccation. We observed development of a spontaneous dry eye phenotype in the CD4-DNTGFβRII mice with aging from 8–14 weeks, with increased corneal surface irregularity, T cell infiltration in the cornea and conjunctiva and corneal barrier disruption. Paradoxically, exposure of 14 week-old mice to a desiccating environment decreased the severity of ocular parameters which was accompanied by decreased CD4^+^ T cell infiltration of the cornea and conjunctiva, increased goblet cell density and decreased levels of T cell related cytokines. RAG1KO recipients of adoptively transferred CD4^+^ T cells from CD4-DNTGFβRII mice subjected to DS developed less severe disease than recipients of CD4^+^ T cells from wild-type mice.

There are several animal models of dry eye currently used throughout the world. They are either spontaneous or induced. Several spontaneous models with lymphocytic infiltration of the lacrimal and salivary glands and ocular surface inflammation mimick Sjögren's syndrome to a certain extent. These include the non-obese diabetic (NOD), MRL/Lpr, NZB/W F1 mouse, and TGF-β1, CD25 and Thrombospodin knock-out (KO) strains [32]–[34].

Similar to the immune phenotype observed in the CD4-DNTGFβRII mice, several other mouse strains (non-obese diabetic, MRL^lpr/lpr^, CD25KO, TGF-β1KO) develop autoimmunity with aging. However, details regarding the ocular surface disease that develops in these models is scarce. TGFβ1KO mice die shortly after birth due to massive, multifocal inflammatory disease [9], [10], [35]. Both lacrimal and submandibular glands of these mice are heavily infiltrated with T cells and show increased levels of T cell related cytokines, notably IFN-γ, IL-1β and TNF-α [10], [35]. The Th-17 signature cytokine IL-17 was not investigated in this model. TGF-β1KO mice develop dry crusty eyes, with lid closure [35]. This may suggest irregularity and dryness of the corneal epithelium that is causing photophobia and irritation. Another model used to study TGF-β1 signaling is the Thrombospondin-1 (TSP1) knock-out strain. TSP-1 is a major physiological activator of latent TGF-β1 [36]. Turpie and colleagues characterized these animals and showed that aging led to progressive lacrimal gland T cell infiltration, a decreased lacrimal gland function measure by peroxidase content (measured ex *vivo*), and an increase in “closed eyes” similar to that seen in the TGF-β1KO mice [36]. Interestingly, similar to our findings in the CD4-DNTGFβRII mice, aged TSP1KO mice had increased corneal surface dye staining, a decreased number of conjunctival goblet cells and increased levels of inflammatory cytokine mRNA transcripts in cornea tissue. These findings indicate that gene deletion or altered TGF-β signaling may compromise generation of natural T regulatory cells (Tregs), leading to an accumulation of auto-reactive, pathogenic CD4^+^ T cells in the lacrimal gland and in the ocular surface tissues with aging. CD4-DNTGFβRII mice have been noted to develop autoreactive CD4^+^ T cells that escape control of T regs [19].

We had developed and characterized an inducible dry eye model where mice subjected to cholinergic blockade and chronically exposed to a drafty environment develop disruption of corneal barrier function, increased production of pro-inflammatory cytokines and metalloproteinases (MMP), activation of mitogen-activated protein kinase (MAPK) intracellular pathways and production of cornified envelope protein precursors [27], [37]–[39] mimicking several features of human dry eye patients [40]–[42]. Upregulation of MAPK pathways requires the full model (air flow, low humidity and scopolamine) [37], [39], [43], [44] and this was not observed by administration of scopolamine alone [43].

Autoimmunity in this model has been demonstrated because adoptive transfer of CD4+T cells primed in vivo in mice subjected to DS were capable of inducing dry eye-changes in the cornea and conjunctiva of immunodeficient recipient mice who were never exposed to dry eye conditions [20], [21], [26].

The role of TGF-β1 in the pathogenesis of dry eye disease is still unclear. Several studies reported increased TGF-β1 protein in tears or mRNA in conjunctival biopsies or increased expression of protein in the conjunctival epithelium and salivary gland biopsies obtained from patients with SS, a severe type of dry eye [2]–[7]. CD4-DNTGFβRII mice show inability to mount a Th-17 response and have been reported to be resistant to development of EAE [12]. To evaluate the contribution of TGF-β1 to the ocular surface immunopathology in dry eye, we challenged CD4-DNTGFβRII mice with a desiccating environment. We have developed and characterized this environmental stress model of dry eye that induces infiltration of the conjunctiva with a mix of Th-17 and Th-1 pathogenic CD4^+^ T cells [6]. We also observed that the pattern of T cell related cytokines in human dry eye patients and mice subjected to the adverse environment are similar, and we have found an increase in expression of matrix metalloproteinases, IL-17A, IFN-γ and TGF-β1, among many other markers. We have also noted increased levels of active TGF-β1 in tears of dry eye patients [5] and in supernatant of cornea and conjunctiva explants obtained from mice subjected to the adverse environment desiccated-stressed (manuscript in preparation). Among human dry eye patients, the highest TGF-β1 activity was found in tears from SS patients [5].

Wild-type C57BL/6 mice when subjected to DS develop a significant uptake of OGD dye, used as a measure of corneal barrier function and a decrease in conjunctival goblet cells similar to what is observed in human dry eye patients. In contrast, CD4-DNTGFβRII mice once stressed showed paradoxical improvement in ocular severity parameters, including corneal barrier function and failure to upregulate Th-1 and Th-17 cytokines. We have previously shown systemic antibody depletion of IL-17A in mice subjected to DS significantly improved corneal barrier function [6] and decreased levels of MMP-9 and MMP-3 transcripts. We have previously reported that dry eye stimulates production of metalloproteinase (MMP-9), as well as other MMPs by the ocular surface epithelia. MMP-9 was found to degrade components of the corneal epithelial basement membrane and tight-junction (TJ) proteins (such as ZO-1 and occludin) that are responsible for maintaining corneal epithelial barrier function [38]. These results indicate that TGF-β1 is critical in the immune response and ocular phenotype observed after desiccating stress; without a proper Th-17 response, there is much less corneal barrier disruption in response to desiccating stress. This also indicates that future therapeutics targeting TGF- β may be successful in controlling dry eye disease in humans. In support of our hypothesis, we have also subjected TSP1KO mice to DS and we observed that they also failed to develop DS-induced corneal barrier dysfunction and loss of conjunctival goblet cells (manuscript in preparation).

Because TGF-β is a pleiotropic cytokine that has multiple functions in the immune system, we then asked if defective TGF-β1 signaling could decrease proliferation of pathogenic CD4^+^ T cells or impair their migration into the ocular surface tissue. Our co-culture experiments showed that CD4^+^ T cells from the CD4-DNTGFβRII mice failed to proliferate when co-cultured with cornea and conjunctiva explants, in contrast to WT cells. Our flow cytometry results showed that CD4-DNTGFβRII mice don't upregulate the chemokine receptor CCR6 in their CD4^+^ T cells after DS. Using an adoptive transfer model, we observed that RAG1KO recipients of CD4^+^ T cells from DS5 CD4-DNTGFβRII mice had significantly higher goblet cell numbers, decreased T cell infiltration in the conjunctiva and lower levels of IL-17A and IFN-γ mRNA in the conjunctiva, indicating that they developed less severe disease. Taken together, these experiments suggest that lack of TGF-β signaling in CD4^+^ T cells after DS decreases both survival and migratory capacity of pathogenic T cells, confirming the critical role of TGF-β1 in inducing ocular surface disease after desiccating stress.

There are certainly limitations to our study. TGF-β is so ubiquitously expressed that genetic deletion approaches in mice are not adequate for studying its blockade due to massive autoimmunity and death [10], [11], [45]. Therefore, alternative models are necessary to study the effect of lack of TGF-β/disruption of TGF-β signaling. We used the CD4-DNTGFβRII strain as a model to study T cell unresponsiveness to TGF-β by creating an experimental dry eye where CD4^+^T cells have been shown to have a pathogenic role [20], [21], [26]. However, one limitation to our study is that these mice also develop systemic disease such as colitis and infiltration of the lungs; despite this multi system inflamamtion, they have been used with success to evaluate the effects of defective TGF-β signaling in generation of Th-17 cells and on disease severity in autoimmune models such as EAE, an animal model of multiple sclerosis [18], [31].

Our results demonstrate for the first time a dual role of TGF-β in the ocular surface. In normal levels, TGF- β supports the generation of regulatory CD4^+^ T cells that supress development of autoimmune reactions over time, while in the stressed setting it supports generation of pathogenic IL-17-producing effector cells.

## Materials and Methods

### Mice

This research protocol was approved by the Baylor College of Medicine Center for Comparative Medicine (animal protocol AN-2032), and it conformed to the standards in the ARVO Statement for the Use of Animals in Ophthalmic and Vision Research. CD4-DNTGFβRII and RAG1KO mice were purchased from The Jackson Laboratory (Bar Harbor, ME) for establishing breeding colonies in our facility. The genotype of CD4-DNTGFβRII mice was confirmed accordingly to the Jackson Labs' protocol (data not shown). Wild-type normal litter mates were used as control. Mice were used at 8 and 14 weeks of age.

### Murine desiccating stress model

DS was induced by subcutaneous injection of scopolamine hydrobromide (0.5 mg/0.2 ml; Sigma-Aldrich, St. Louis), QID (08:00, 12:00, 14:00, and 17:00 h), for 5 or 10 consecutive days (DS5 or DS10, respectively) in CD4-DNTGFβRII and WT mice at 14 weeks of age, as previously published [6], [23], [26]. Mice were placed in a cage with a perforated plastic screen on one side to allow airflow from a fan placed six inches in front of it for 16 h/day. Room humidity was maintained at 30–35%. Control mice were maintained in a non-stressed (NS) environment containing 50–75% relative humidity without exposure to forced air.

Forty five WT and CD4-DNTGFβRII mice, of both genders, were used at 8 weeks of age. Seventy five WT and CD4-DNTGFβRII mice, of both genders, were used at 14 weeks of age per time point (NS, DS5): five mice for histologic sections, eight mice for gene analysis, twenty mice for flow cytometry, and twelve mice for corneal staining, twenty mice for the co-culture experiments and ten mice for the adoptive transfer. Evaluation of corneal smoothness was performed immediately after the euthanasia, on the same mice that were used for evaluating gene expression and histology. Ten RAG1KO mice were recipient of NS and DS5 mice of both strains.

### RNA isolation and Real time PCR

Total RNA from the conjunctiva collected was extracted using a PicoPure RNA isolation® Kit (Arcturus, Applied Biosystems, Foster City, CA) according to the manufacturer's instructions, quantified by a NanoDrop® ND-1000 Spectrophotometer (Thermo scientific, Wilmington, DE) and stored at −80°C. Eight samples per group/strain were used, and 1 sample consisted of pooled eyes of the same animal. Samples were treated with DNAse to prevent genomic DNA contamination, according to the manufacturer's instructions (Qiagen, Valencia, CA). The RNA concentration was measured by its absorption at 260 nm and samples were stored at −80°C until use.

First-strand cDNA was synthesized with random hexamers by M-MuLV reverse transcription (Ready-To-Go You-Prime First-Strand Beads; GE Healthcare, Inc., Arlington Heights, NJ), as previously described [6]. Real-time PCR was performed with specific MGB probes (Taqman; Applied Biosystems, Inc. [ABI], Foster City, CA) and PCR master mix (Taqman Gene Expression Master Mix), in a commercial thermocycling system (Mx3005P QPCR System; Stratagene, La Jolla, CA), according to the manufacturer's recommendations. Murine MGB probes were GAPDH (Mm99999915), MMP-3 (Mm00440295), MMP-9 (Mm00442991),IL-6 (Mm00446490), IL-17A (Mm00439619), IFN-γ (Mm00801778), IL-13 (Mm00434165), IL-23(Mm00518984), RORγT (Mm00441139), T-bet (Mm00450960) and GATA-3 (Mm00484683). The GAPDH gene was used as an endogenous reference for each reaction. The results of quantitative PCR were analyzed by the comparative C_T_ method where target change = 2^−^ 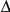 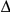 ^C^_T_. The results were normalized by the C_T_ value of GAPDH. When comparing both strains, the NS WT at 14 weeks of age served as the calibrator. In the desiccating stress model, the mean C_T_ of relative mRNA level in the non-stressed control group of each strain was used as the calibrator.

### Histology and Periodic Acid Schiff Staining

Goblet cell density was evaluated in NS WT and CD4-DNTGFβRII at 8 and 14 weeks of age and DS5 mice of both strain (n = 5). Eyes and ocular adnexa were surgically excised, fixed in 10% formalin, paraffin embedded and 8 µm sections were cut. Ocular sections were cut at the center of the eye, where the lens has its maximum diameter. Sections were stained with periodic-Schiff (PAS) reagent for measuring goblet cell density and were examined and photographed with a microscope equipped with a digital camera (Eclipse E400 with a DS-Fi1; Nikon). The number of goblet cells in the superior and inferior conjunctiva was measured in 3 sections from each eye that were 300 µm apart from each other, using image-analysis software (NIS Elements Software, version 3.0, BR, Nikon) and expressed as number of goblet cells per mm.

For immunohistochemistry, the eyes and adnexa of mice/time point (n = 5) were excised, embedded in optimal cutting temperature (OCT compound; VWR, Suwanee, GA), and flash frozen in liquid nitrogen. Sagittal 8-µm sections were cut with a cryostat (HM 500; Micron, Waldorf, Germany) and placed on glass slides that were stored at −80°C.

### Immunohistochemistry

Immunohistochemistry was performed to detect and count the cells in the cornea and conjunctival epithelium and stroma that stained positively for CD4 (clone H129.9, 10 µg/mL, BD Bioscience, San Diego, CA) and CD8α (clone 53e6.7, 3.125 µg/mL, BD Bioscience). Cryosections were stained with these primary antibodies and appropriate biotinylated secondary antibodies (BD Pharmingen; Jackson Immune Laboratories, West Grove, PA) and Vectastain Elite ABC using NovaRed reagents (Vector, Burlingame, CA), as previously described [22]. Secondary antibody alone and appropriate anti-mouse isotype (BD Biosciences) controls were also performed. Three sections from each animal were examined and photographed with a microscope equipped with a digital camera (Eclipse E400 with a DS-Fi1; Nikon). Positively stained cells were counted in the goblet cell rich area of the conjunctiva, over a length of at least 500 µm in the epithelium for a distance of 500 µm using image-analysis software (NIS Elements Software, version 3.0, BR, Nikon).

### Corneal Permeability

Corneal epithelial permeability to Oregon green dextran (OGD; 70,000 molecular weight [MW]; Invitrogen, Eugene, OR) was assessed in the nonstressed WT and CD4-DNTGFβRII at 8 and 14 weeks of age (12 mice/group/time point, in three sets of experiments) as previously described [6], with a minor modification. Briefly, 0.5 µL of 50 mg/mL OGD was instilled onto the ocular surface 1 minute before euthanasia. Corneas were rinsed with PBS and photographed with a high dinamic and resolution digital camera (Coolsnap HQ2, Photometrics, Tucson, AZ) attached to a stereoscopic zoom microscope (SMZ 1500; Nikon, Melville, NY), under fluorescence excitation at 470 nm. The severity of corneal OGD staining was graded in digital images by masked 2 observers, using NIS Elements (version 3.0, Nikon, Melville, NY) within a 2-mm diameter circle placed on the central cornea. The mean fluorescene intensity measured by the software inside this central zone was transferred to a database and the results averaged within each group. Results are presented as mean±standard deviation of gray levels.

### Evaluation of Corneal Smoothness

Corneal smoothness was assessed in 26 eyes of the nonstressed WT and CD4-DNTGFβRII at 8 and 14 weeks of age (13 mice/group/time point, in two sets of experiments) [22]. Smoothness of images taken by a stereoscopic zoom microscope fiber optic ring illuminator (SMZ 1500; Nikon) reflected off the corneal surface was graded in digital images by two masked observers and averaged within each group. The corneal irregularity severity score was calculated using a 5-point scale based on the number of distorted quadrants in the reflected ring as previously described [22]. Results are presented as mean±standard deviation.

### Flow Cytometry analysis of murine cells

Freshly isolated cells from cornea, conjunctiva and cervical lymph nodes were stained with anti-CD16/32 (to block Fc receptors, BD Pharmigen, San Diego, CA), followed by cell surface staining with FITC-CD4, PE-anti-CD8, APC-anti-CCR6 (from BD Pharmigen, San Diego,CA), orAPC-anti-CXCR3 (R&D Systems, Minneapolis). Positive controls consisted of splenocytes processed at the same time under the same protocol. Negative controls consisted of cells stained with FITC, PE or APC labeled isotype antibodies (BD Pharmigen). Cells were resuspended in violet dye (live/dead cell fixable staining, Invitrogen-Molecular Probes, Carlsbad, CA) and washed. Cells were then resuspended in fixation-permeabilization solution (Cytofix/Cytoperm; BD Pharmingen) and stored at 4°C until the next day when the analysis was performed. A BD LSRII Benchtop cytometer was used for flow cytometry and data were analyzed using BD Diva Software (BD Pharmigen).

### Isolation of murine CD4^+^ T cells and adoptive transfer

Superior CLN and spleens from donor mice (NS and DS5 WT and CD4-DNTGFβRII at 14 weeks of age) and were meshed gently between two frosted end glass slides, as previously described [26]. Untouched CD4+ cells were isolated using magnetic beads according to the manufacturer's instructions (MACS system; Miltenyi Biotec). One donor-equivalent of cells were transferred intraperitoneally (i.p.) to T cell deficient mice (RAG1KO). One donor-equivalent is defined as the number of cells remaining after the respective *in vitro* manipulation (e.g., CD4^+^ T cells) of a single set of lymph nodes or spleen (approximately 5×10^6^ CD4^+^ cells). The adoptive transfer recipients were sacrificed 72 hours after the initial adoptive transfer. In some experiments, eyes were collected for histology, while in others cornea and conjunctiva were processed for RNA analysis.

### Co-culture and proliferation assays

Four cornea and conjunctiva explants/well excised from NS and DS5 WT mice were co-culture with 0.5×10^6^ non-stressed CD4^+^ T cells isolated from WT and CD4-DNTGFβRII at 14 weeks of age. Four days after co-culture, the water soluble tetrazolium (WST, Chemicon International, Temecula, CA) assay was performed according to the manufacturer's instructions. Absorbance readings were recorded using Spectra Max 190 at 440 nm (Molecular Devices, Sunnydale, CA). The assay was performed twice and each group consisted of four biological replicates/experiment. Results presented as percentage change in proliferation within each strain using NS CD4+T cells as the calibrator.

### Statistical analysis

The normality of data was checked with the Kolmogorov-Smirnov test using the Dallal and Wilkinson approximation. Kruskall-Wallis test (multiple comparison test) was used to determine overall statistical significance followed by a two-tailed T-test for individual differences in NS WT and CD4-DNTGFβRII at 8 weeks, and CD4-DNTGFβRII and WT before and after DS (within strain comparison), using p≤0.05 as statistically significant. These tests were performed using GraphPad Prism 5.0 software (GraphPad Software Incorporation, San Diego, CA).

## References

[1] Li MO, Wan YY, Sanjabi S, Robertson AK, Flavell RA (2006). Transforming growth factor-beta regulation of immune responses.. Annu Rev Immunol.

[2] Gupta A, Monroy D, Ji Z, Yoshino K, Huang AJW (1996). Transforming growth factor beta-1 and beta-2 in human tear fluid.. Curr Eye Res.

[3] Yoshino K, Garg R, Monroy D, Ji Z, Pflugfelder SC (1996). Production and secretion of transforming growth factor beta (TGF-β) by the human lacrimal gland.. Curr Eye Res.

[4] Pflugfelder SC, Jones D, Ji Z, Afonso A, Monroy D (1999). Altered cytokine balance in the tear fluid and conjunctiva of patients with Sjogren's syndrome keratoconjunctivitis sicca.. Curr Eye Res.

[5] Zheng X, de Paiva CS, Rao K, Li DQ, Farley WJ (2010). Evaluation of the transforming growth factor-beta activity in normal and dry eye human tears by CCL-185 cell bioassay.. Cornea.

[6] de Paiva CS, Chotikavanich S, Pangelinan SB, Pitcher JI, Fang B (2009). IL-17 disrupts corneal barrier following desiccating stress.. Mucosal Immunology.

[7] Sun D, Emmert-Buck MR, Fox PC (1998). Differential cytokine mRNA expression in human labial minor salivary glands in primary Sjogren's syndrome.. Autoimmunity.

[8] Rahimy E, Pitcher JD, Pangelinan SB, Chen W, Farley JW (2010). Spontaneous Autoimmune Dacryoadenitis in Aged CD25KO.. Am J Pathol.

[9] Shull MM, Ormsby I, Kier AB, Pawlowski S, Diebold RJ (1992). Targeted disruption of the mouse transforming growth factor-beta 1 gene results in multifocal inflammatory disease.. Nature.

[10] McCartney-Francis NL, Mizel DE, Redman RS, Frazier-Jessen M, Panek RB (1996). Autoimmune Sjogren's-like lesions in salivary glands of TGF-beta1-deficient mice are inhibited by adhesion-blocking peptides.. J Immunol.

[11] Nandula SR, Amarnath S, Molinolo A, Bandyopadhyay BC, Hall B (2007). Female mice are more susceptible to developing inflammatory disorders due to impaired transforming growth factor beta signaling in salivary glands.. Arthritis Rheum.

[12] Veldhoen M, Hocking RJ, Flavell RA, Stockinger B (2006). Signals mediated by transforming growth factor-beta initiate autoimmune encephalomyelitis, but chronic inflammation is needed to sustain disease.. Nat Immunol.

[13] Wan YY, Flavell RA (2007). ‘Yin-Yang’ functions of transforming growth factor-beta and T regulatory cells in immune regulation.. Immunol Rev.

[14] Bettelli E, Oukka M, Kuchroo VK (2007). T(H)-17 cells in the circle of immunity and autoimmunity.. Nat Immunol.

[15] Veldhoen M, Hocking RJ, Atkins CJ, Locksley RM, Stockinger B (2006). TGFbeta in the context of an inflammatory cytokine milieu supports de novo differentiation of IL-17-producing T cells.. Immunity.

[16] Bettelli E, Carrier Y, Gao W, Korn T, Strom TB (2006). Reciprocal developmental pathways for the generation of pathogenic effector TH17 and regulatory T cells.. Nature.

[17] Mangan PR, Harrington LE, O'Quinn DB, Helms WS, Bullard DC (2006). Transforming growth factor-beta induces development of the T(H)17 lineage.. Nature.

[18] Gorelik L, Flavell RA (2000). Abrogation of TGFbeta signaling in T cells leads to spontaneous T cell differentiation and autoimmune disease.. Immunity.

[19] Fahlen L, Read S, Gorelik L, Hurst SD, Coffman RL (2005). T cells that cannot respond to TGF-beta escape control by CD4(+)CD25(+) regulatory T cells.. J Exp Med.

[20] Zhang X, Chen W, de Paiva CS, Corrales RM, Volpe EA (2011). Interferon-gamma exacerbates dry eye-induced apoptosis in conjunctiva through dual apoptotic pathways.. Invest Ophthalmol Vis Sci.

[21] Zhang X, Chen W, de Paiva CS, Volpe EA, Gandhi NB (2011). Desiccating Stress Induces CD4(+) T-Cell-Mediated Sjogren's Syndrome-Like Corneal Epithelial Apoptosis via Activation of the Extrinsic Apoptotic Pathway by Interferon-gamma.. Am J Pathol.

[22] de Paiva CS, Corrales RM, Villarreal AL, Farley W, Li DQ (2006). Apical corneal barrier disruption in experimental murine dry eye is abrogated by methylprednisolone and doxycycline.. Invest Ophthalmol Vis Sci.

[23] de Paiva CS, Corrales RM, Villarreal AL, Farley WJ, Li DQ (2006). Corticosteroid and doxycycline suppress MMP-9 and inflammatory cytokine expression, MAPK activation in the corneal epithelium in experimental dry eye.. Exp Eye Res.

[24] Ecoiffier T, El AJ, Rashid S, Schaumberg D, Dana R (2008). Modulation of integrin alpha4beta1 (VLA-4) in dry eye disease.. Arch Ophthalmol.

[25] Lee HS, Chauhan SK, Okanobo A, Nallasamy N, Dana R (2011). Therapeutic Efficacy of Topical Epigallocatechin Gallate in Murine Dry Eye.. Cornea.

[26] Niederkorn JY, Stern ME, Pflugfelder SC, de Paiva CS, Corrales RM (2006). Desiccating Stress Induces T Cell-Mediated Sjogren's Syndrome-Like Lacrimal Keratoconjunctivitis.. J Immunol.

[27] Corrales RM, Stern ME, de Paiva CS, Welch J, Li DQ (2006). Desiccating stress stimulates expression of matrix metalloproteinases by the corneal epithelium.. Invest Ophthalmol Vis Sci.

[28] Chotikavanich S, de Paiva CS, Li DQ, Chen JJ, Bian F (2009). Production and Activity of Matrix Metalloproteinase-9 on the Ocular Surface Increase in Dysfunctional Tear Syndrome.. Invest Ophthalmol Vis Sci Feb 28. [Epub ahead of print].

[29] Gao J, Siemasko KF, Niederkorn JY, Calder VL, Calonge M (2008). Corneas Exposed to Desiccating Stress Are Immunogenic and Induce T Cell Proliferation in Mice With Experimental Lacrimal Keratoconjunctivitis (LKC).. Invest Ophthalmol Vis Sci 2008.

[30] Cox CA, Shi G, Yin H, Vistica BP, Wawrousek EF (2008). Both Th1 and Th17 are immunopathogenic but differ in other key biological activities.. J Immunol.

[31] Wang C, Kang SG, Lee J, Sun Z, Kim CH (2009). The roles of CCR6 in migration of Th17 cells and regulation of effector T-cell balance in the gut.. Mucosal Immunol.

[32] Van Blokland SC, Versnel MA (2002). Pathogenesis of Sjogren's syndrome: characteristics of different mouse models for autoimmune exocrinopathy.. Clin Immunol.

[33] de Paiva CS, Hwang CS, Pitcher JD, Pangelinan SB, Rahimy E (2010). Age-related T-cell cytokine profile parallels corneal disease severity in Sjogren's syndrome-like keratoconjunctivitis sicca in CD25KO mice.. Rheumatology (Oxford).

[34] Yang Z, Mu Z, Dabovic B, Jurukovski V, Yu D (2007). Absence of integrin-mediated TGFbeta1 activation in vivo recapitulates the phenotype of TGFbeta1-null mice.. J Cell Biol.

[35] McCartney-Francis NL, Mizel DE, Frazier-Jessen M, Kulkarni AB, McCarthy JB (1997). Lacrimal gland inflammation is responsible for ocular pathology in TGF-beta 1 null mice.. Am J Pathol.

[36] Turpie B, Yoshimura T, Gulati A, Rios JD, Dartt DA (2009). Sjogren's syndrome-like ocular surface disease in thrombospondin-1 deficient mice.. Am J Pathol.

[37] de Paiva CS, Pangelinan SB, Chang E, Yoon KC, Farley WJ (2009). Essential role for c-Jun N-terminal kinase 2 in corneal epithelial response to desiccating stress.. Arch Ophthalmol.

[38] Pflugfelder SC, Farley W, Luo L, Chen LZ, de Paiva CS (2005). Matrix metalloproteinase-9 knockout confers resistance to corneal epithelial barrier disruption in experimental dry eye.. Am J Pathol.

[39] Luo L, Li DQ, Corrales RM, Pflugfelder SC (2005). Hyperosmolar saline is a proinflammatory stress on the mouse ocular surface.. Eye & Contact Lens.

[40] Chotikavanich S, de Paiva CS, Li DQ, Chen JJ, Bian F (2009). Production and activity of matrix metalloproteinase-9 on the ocular surface increase in dysfunctional tear syndrome.. Invest Ophthalmol Vis Sci.

[41] Solomon A, Dursun D, Liu Z, Xie Y, Macri A (2001). Pro- and anti-inflammatory forms of interleukin-1 in the tear fluid and conjunctiva of patients with dry-eye disease.. Invest Ophthalmol Vis Sci.

[42] Lam H, Blieden L, de Paiva CS, Farley WJ, Stern ME (2008). Tear Cytokine Profiles in Dysfunctional Tear Syndrome.. Am J Ophthalmol Nov 5.

[43] Luo L, Li DQ, Doshi A, Farley W, Corrales RM (2004). Experimental dry eye stimulates production of inflammatory cytokines and MMP-9 and activates MAPK signaling pathways on the ocular surface.. Invest Ophthalmol Vis Sci.

[44] de Paiva CS, Corrales RM, Villarreal AL, Farley WJ, Li DQ (2006). Corticosteroid and doxycycline suppress MMP-9 and inflammatory cytokine expression, MAPK activation in the corneal epithelium in experimental dry eye.. Exp Eye Res.

[45] Cohen MS, Shorr N (1992). Eyelid reconstuction with hard palate mucosa grafts.. Ophthal Plast & Reconstr Surg.

